# Nutritional Challenges in Nursing Homes: Pilot Study on Macronutrient Intake and Status of Vitamins D and B12

**DOI:** 10.3390/nu16101495

**Published:** 2024-05-15

**Authors:** Živa Lavriša, Igor Pravst

**Affiliations:** 1 Nutrition and Public Health Research Group, Nutrition Institute, Koprska ulica 98, SI-1000 Ljubljana, Slovenia; ziva.lavrisa@nutris.org; 2 Biotechnical Faculty, University of Ljubljana, Jamnikarjeva 101, SI-1000 Ljubljana, Slovenia; 3 VIST–Faculty of Applied Sciences, Gerbičeva cesta 51A, SI-1000 Ljubljana, Slovenia

**Keywords:** dietary intake, nursing home, nutrition, older adults

## Abstract

Older adults living in nursing homes (NH) are considered a population group that could be at risk in terms of nutrition, even more so than their community-dwelling peers. Evidence on the nutritional status of NH residents is scarce, as they are commonly excluded from population-based dietary studies. This is also the case in Slovenia. In the presented pilot study, we assessed the intake of macronutrients as well as the intake and status of vitamin D and vitamin B12 on a sample of NH and NH daycare center users to explore the need for a larger representative study. The pilot study included 37 participants from three Slovenian NH (20 participants) and their daycare centers (17 participants). Daycare centers offer daytime care services for older adults, where users are also provided with major meals during their stay. Intakes of energy and nutrients were estimated by three 24 h dietary records. Fasting blood samples were collected for the assessment of vitamin D and vitamin B12 status. Over 90% of the participants had daily energy and protein intakes below recommendations (reference values: energy intake: males 2100 kcal and females 1700 kcal; protein intake > 1 g/kg body mass). The males’ median daily intakes of vitamin D were 1.7 µg (1.5 µg females), and 2.3 µg for vitamin B12 (2.0 µg females). None of the participants had adequate vitamin D intake (>20 µg), and 92.3% males and 87.5% females had inadequate vitamin B12 intake (<4 µg). The prevalence of vitamin D deficiency (serum 25-OH-D conc. < 30 nmol/L) was 100% among NH residents and 53% among NH daycare center users. The prevalence of vitamin B12 deficiency was found in 20% of NH residents. The study results highlighted that certain nutrients might be critical in this population, especially among NH residents; however, a more thorough investigation with the inclusion of other important markers of nutritional status should be performed on a larger, representative sample to support the development and implementation of appropriate public health interventions.

## 1. Introduction

Appropriate nutrition is essential for maintaining overall health and wellness in all life stages but is considered particularly important in the older population (people aged 65 years and older). Various physiological changes and medical conditions [1] occurring at an older age can affect an individuals’ ability to consume, absorb, and metabolize nutrients [2]. Among older adults, those living in nursing homes (NH) have been identified as a population group, particularly at risk [3,4,5]. The prevalence of malnutrition in NH is generally higher than in the community-dwelling older population and is associated with health risks such as sarcopenia, cognitive impairment, cardiovascular disorders [6], and higher mortality rates [3,6,7]. In NH, reported rates of malnutrition and inadequate nutrient intakes can reach up to 50% or even higher [8], but for various reasons, such cases often remain unrecognized and untreated [9]. A suboptimal intake of specific (micro)nutrients can occur even in cases where energy intakes are generally sufficient, particularly vitamin B12, vitamin D, depending on the individual’s diet [10]. In older adults, protein is considered a macronutrient of particular concern; a notable proportion of institutionalized older adults do not meet their daily protein needs [11]. Adequate dietary protein intake (a minimum of 1 g/kg body mass daily, depending on the individual’s needs and health status) is also one of several important factors in the prevention of sarcopenia, which is defined as a loss of muscle strength and muscle mass, as adequate protein intake helps to maintain muscle mass [12,13]. Besides low energy and protein intake, studies on nutrition in NH often expose high fat intake [14] and low intake of carbohydrates; furthermore, less refined foods would also contribute to a more favorable intake of dietary fibers [15,16].

Micronutrients play a crucial role in maintaining health and well-being, especially in older adults. Certain micronutrients such as calcium and iron are important for older adults, as they play essential roles in bone health, oxygen transport, and overall physiological function, addressing concerns such as osteoporosis, anemia, and maintaining energy levels [17]. We specifically chose to analyze vitamin D and vitamin B12 status. We were able to provide blood biomarker analysis for these two, and moreover, both vitamin D and vitamin B12 deficiencies are prevalent among older adults and can have significant health implications, including increased risk of falls, fractures, anemia, and cognitive decline [6]. Although insufficient vitamin D intake and status are common in all population groups in Europe [18], the reported prevalence of vitamin D deficiency is particularly high in NH residents, where this is often unrecognized and untreated [19]. Also, NH residents are often less exposed to the sun than their community-dwelling peers, which also affects their vitamin D status [20]. In older adults, vitamin D, together with calcium, is important for the prevention of falls and fractures through its role in the maintenance of bone density and muscle function [21,22]. Besides vitamin D status, maintaining proper vitamin B12 status can also be challenging in older adults (65 years and older) due to impaired absorption caused by gastric abnormalities that progress with age, medication, and other physiological changes [23], particularly affecting older men [24,25,26]. In such cases, solely food sources of vitamin B12, such as foods of animal origin, are not sufficient to cover the needs, although these are often consumed in excessive amounts, particularly in older males [27]. In certain countries (e.g., the United States of America), vitamin B12 and vitamin D supplementation is therefore already advised for older adults [28].

Diet-related risk factors in Slovenia have been investigated in a national study, SI.Menu [27], which included older adults (416 participants, 65 years and older) but focused only on community-dwelling older adults, excluding those living in NH. Nevertheless, a notable proportion of the community-dwelling older adults (21% of men and 43% of women) had protein intake below recommended (<1.0 g per kg of body weight) [27]. Furthermore, a high prevalence (35%) of winter vitamin D deficiency has been observed [29], while the prevalence of vitamin B12 deficiency was 7% [24]. Studies performed on NH populations in other countries showed that low energy and protein intake were often problematic, and vitamin D deficiency was seen as particularly concerning [20,30,31].

Our aim was to provide the first insights on nutrition and certain nutrients in older adults living in Slovenian nursing homes and to assess the possible need for a larger study on this population group, as currently such data are not available. The hypothesis of the present study was that energy and macronutrient daily intakes, as well as intake and status of vitamin B12 and vitamin D, are expected to be lower than recommendations. We investigated the daily intake of energy and selected nutrients. Vitamin D and vitamin B12 status were assessed using established blood biomarkers. The present pilot study lays the groundwork for investigating this topic on a larger scale.

## 2. Methods

The pilot study was carried out using initial screening data collected in the scope of an intervention study, which then further explored how to improve nutritional status of older adults in nursing homes. The study was conducted in accordance with the Declaration of Helsinki, approved by the Institutional Ethics Committee of VIST–Faculty of Applied Sciences (Approval No. 2020/1-ET-SK; 18 February 2020), and included in the ClinicalTrials.gov register under the entry NCT05661006, where the protocol details can be found [32]. The study was conducted in three Slovenian nursing homes and their NH daycare centers in different statistical regions (a geographical division used for statistical and administrative purposes), Ptuj, Rogaška Slatina and Novo Mesto, which were willing to participate in the pilot study. In the nursing homes, residents are supplied with all foods (but are free to complement their diet with other foods, i.e., those purchased in markets or supplied by relatives). On the other hand, NH daycare center users are not permanent residents of NHs and sleep at home; in the NH, they were supplied with at least two major meals, including lunch, on all days of the week. The participants in the study were recruited based on an invitation that was published in participating NH facilities, using a first-come, first-served approach. The inclusion criteria were: at least 65 years old; a body mass index lower than 32 kg/m^2^; and no protein, vitamin D, or vitamin B12 supplement used during the past two months. The data were collected between February and March 2020. The participants were aged between 65 and 92 years. For the determination of body mass index (BMI), participants’ body weight and height were measured by Seca 799 medical scale (Seca GmbH, Hamburg, Germany). Data on food intake were collected using three 24 h dietary records (on two weekdays and one weekend day). All meals were recorded, regardless of the place offered. The data analysis of nutrient intakes was calculated from an average of the three dietary records. Participants were cognitively capable to fill out the records by themselves, with some help from nursing home staff. Participants recorded all foods that they consumed in a specific day (i.e., foods provided by NHs, purchased in markets, provided by relatives, etc.), regardless of place of consumption, along with the portion sizes. Portion sizes were estimated with the support of a nationally validated picture book [33] containing commonly consumed foods and simple dishes in different portion sizes. If a participant had issues stating the portion size, they were able to look into the picture book for a reference on the portion size. Average habitual nutrient intakes were calculated by trained researchers from the dietary records using the Open Platform for Clinical Nutrition (OPEN) [34], which includes the Slovenian food composition database. Energy and nutrient intakes were compared to Slovenian national dietary reference values for older adults: energy intake (predisposition to low physical activity): 2100 kcal/day (males), 1700 kcal (females); protein: at least 1 g/kg body weight/day; carbohydrates: >50% energy intake; fat: 30% energy intake; vitamin D (20 µg) and B12 (4 µg) [35].

Vitamin D and vitamin B12 status were assessed using blood biomarkers. Fasting blood samples were collected by NH nurses for the laboratory determination of the concentration of serum 25-hydroxy-vitamin D (25-OH-D) and vitamin B12. The blood analyses were carried out in the accredited medical diagnostic laboratory Adrialab/Synlab (Ljubljana, Slovenia). For the quantitative determination of 25-OH-D in human serum, an architect 25-OH-D (Abbott Ireland, Longford, Ireland) chemiluminescent microparticle immunoassay was used. The correlation coefficient with the ID-LC-MS/MS method within the assay’s measuring interval (12–378 nmol/L) was r = 0.99 (95% CI: 0.99, 1.05). Quantitative determination of serum vitamin B12 concentration was carried out using an intrinsic factor chemiluminescent microparticle immunoassay on the Alinity analyser (Abbott Ireland, Longford, Ireland). The following cut-offs were used in the evaluation: Vitamin D deficiency–serum 25-OH-D concentration below 30 nmol/L (12 µg/L); insufficient vitamin D status–serum 25-OH-D concentration below 50 nmol/L (20 µg/L); and optimal vitamin D status–serum 25-OH-D concentration above 75 nmol/L (30 µg/L) [36,37,38]. Serum vitamin B12 concentration below 148 pmol/L was considered a B12 deficiency, while a concentration of 148–221 pmol/L was considered a “low vitamin B12 concentration”, and levels above 221 pmol/L were considered adequate vitamin B12 status [39,40].

The present pilot study was not powered to present nationally representative data but to offer an insight into potential nutrition-related issues. The median and percentile values were reported for energy and nutrient intakes, as well as for serum concentrations of 25-OH-D and vitamin B12. The adherence to reference values for energy and nutrient intakes, and reference values for biomarkers of vitamin D and vitamin B12 status were reported as prevalence (%) in terms of quartile distribution for each sex and subgroup. The distribution of the key outcomes was not normal. As the sample size was small and the participants’ characteristics were diverse regarding the observed outcomes and potential clustering within the samples, we provided descriptive statistics. For data analyses, IBM SPSS Version 27 (IBM SPSS, IBM Corp., Armonk, NY, USA) was used.

## 3. Results

Altogether, 40 participants were initially included in the screening, and 37 (24 females and 13 males) completed the screening phase and were included in the study. Three participants withdrew from the study soon after signing the informed consent, and their data provided were incomplete; therefore, they were excluded from the analyses. Of the 37 participants enrolled in the study, 20 were permanent NH residents (10 males, 10 females), while 17 were recruited from NH daycare centers (3 males, 14 females). The mean age of participants was 75 years, with a standard deviation (SD) of 8.9, mean height was 164.7 cm (SD: 9.9), and mean weight was 76.8 kg (SD: 11.7).

Over 90% of the participants had daily energy intakes below the recommendations (Table 1). The mean protein intakes of the participants were also not compliant with recommendations; the median protein intake/kilogram of body mass/day was 0.6 for males and 0.8 for females, and 92% of the participants of both sexes did not reach the recommended daily protein intake for older adults (1 g/kg body mass). Approximately half of males and females met the recommended carbohydrate daily intake, which should exceed 50% of daily energy intake. Around 80% of participants of both sexes had daily fat intake above 30% TEI (total energy intake), trending toward a higher overall total energy intake from fats than recommended (interquartile range (IQR) 25 = 31% of TEI males, IQR25 = 32% of TEI females). An analysis of the nutrient intakes of participants, divided into NH residents and daycare center users (Table 2), showed that around 95% of NH residents and daycare center users had lower energy intakes than recommended. Comparing NH residents and NH daycare center users, it is interesting that in NH residents, 85% of participants had carbohydrate intakes above 50% TEI, while in NH daycare center users, this was noted in only 18% of participants.

Both vitamin D and vitamin B12 daily intakes were below the recommendations in both males and females, as well as NH residents and NH daycare center users (Table 1 and Table 2). The median daily vitamin D intake was 1.7 µg in males and 1.5 µg in females, while for NH daycare center users it was 1.5 µg and 2.6 µg, respectively. For vitamin B12, 92% of NH residents and 88% of NH daycare center users had lower daily intakes than recommended, and both NH residents and NH daycare center users had suboptimal vitamin D intake. All the permanent residents of NH had vitamin D deficiency, while among NH daycare center users, the prevalence of deficiency was about 53% (Table 3). Furthermore, 20% of NH residents and 35.3% of NH daycare center users were vitamin B12 deficient, with serum concentrations below 148 pmol/L. The prevalence of both low serum B12 concentration and deficiency was more notable in males than in females.

## 4. Discussion

The results of the pilot study highlighted diet-related challenges for older adults living in NH, which require further investigation and action. Among the studied parameters, energy intake as well as vitamin D and vitamin B12 were found to be potential concerns in this population. In Slovenia, about 460,000 people are aged 65 or older, and 18,000 live in nursing homes. It is known that excess or deficiency of energy and/or nutrients leads to negative health consequences [41,42]. Among older adults, particularly those in different care settings, inadequate protein and energy intake is highly prevalent, even up to 85% [43], which can result in frailty and loss of muscle mass and function [44]. In the present pilot study, most of the participants, regardless of sex, had suboptimal daily energy and protein intake, as well as vitamin D and vitamin B12. The reported daily energy intakes in our study were considerably lower than the recommended values for both sexes. A significant proportion of participants of both sexes (above 90%) had inadequate energy intakes, while 92% of males and females had insufficient protein intakes. This finding is consistent with other studies, which report similar issues in this population [45,46,47]. It should be noted that our reported daily energy and nutrient intakes correspond to habitual intakes for the average of three days of assessment (for which 24 h food records were available), and are not directly comparable with the usual (long-term) intakes, which were estimated in the national SI.Menu study for community-dwelling older adults in Slovenia [27]. Nevertheless, the difference in energy intakes in the SI.Menu study (2291 kcal for males and 1758 kcal for females) cannot be explained only by the methodological differences between the two studies.

It is well established that in addition to energy intake, protein intake is also challenging for the older population, although proteins are crucial for the maintenance and function of muscle mass, which notably affects quality of life [48]. In older adults, protein requirements are higher due to various physiological changes, inflammation, and medical conditions [49]. The PROT-AGE study group set a recommendation of average daily protein intake for older adults of at least 1.0–1.2 g of protein per kilogram of body mass [50], while a threshold of daily intake of 1 g of protein per kg of body mass was adopted in the Slovenian national recommendation of nutrient intakes for older adults [35]. We observed daily protein intakes per kg of body mass below recommended amounts in both sexes, which shows a need for intervention, which would optimally be based on individual requirements. The mean daily protein intake in our study (59.5 g for males and 51.4 g for females) was very similar to the findings of a German study on NH residents (59 g/day for males, 49 g for females) [51]. A Dutch study [52] reported a daily protein intake of 58 g for NH residents. On the other hand, a study in Spanish NH found considerably higher daily protein intakes of 83 g [53], suggesting that more protein is available from the NH menus for Spanish residents. Overall, research shows that the daily protein intakes of NH residents are generally lower than those of community-dwelling older adults, for which there might be different reasons, which could be further explored [7]. For example, the usual daily protein intakes of Slovenian older adults living in the community in the SI.Menu study were 105 g (1.3 g per kg of body weight) for males, and 81 g (1.1 g per kg of body weight) for females, while protein intake requirements were not met for 21% and 43%, respectively [27]. When looking at NH residents and daycare center users separately, the trend showed a higher daily protein intake was observed in NH daycare center users, who do not exclusively depend on NH meals. On the other hand, this pilot study highlighted better results for daily carbohydrate intake in NH compared to NH daycare center users; however, the in-depth statistics that would highlight such differences were not performed. Conversely, the majority of community-dwelling older adults in Slovenia (84% of males and 64% of females) did not meet these recommendations [27]. The observed fat intake was high, exceeding 30% of energy intake in the majority of males and females in all NH daycare center users; however, this was the case for 65% of NH residents. Other studies in such populations have also reported a high fat intake [6], while only about a quarter of community-dwelling older adults (26% of males, 21% of females) had more than 30% of their energy intake from fats [27]. Body mass index showed a similar trend between NH residents and daycare center users in Slovenia; however, it was higher in NH residents. Carbohydrate intake showed a more concerning trend in daycare center users, as most participants did not reach the recommended 50% of daily energy intake from this source. We also focused on selected micronutrients, namely vitamin D and vitamin B12. A longitudinal SENECA study (Survey in Europe on Nutrition and the Older adults; a Concerted Action) highlighted that as many as a quarter of the community-dwelling males and half of the females included in the study had low daily dietary intake or low serum concentrations of vitamin B12 and vitamin D [54]. In our study, the daily dietary intakes for both vitamin D and vitamin B12 were below the recommendations for all groups. It should be noted that the observed intakes reflect only intake from food because food supplement users were excluded from the study. For both vitamins, and especially vitamin D, the trend shows higher intake in the subpopulation group of NH daycare center users than NH residents. Low intakes of these vitamins were also reflected in the levels of blood biomarkers. The observed prevalence of vitamin D deficiency was lower in NH daycare center users. This could also be due do different lifestyle habits in this population, that affect the biosynthesis of vitamin D due to sun exposure.

Our most alarming observation was that all (100%) of the participants who were permanent NH residents had vitamin D deficiency, with serum 25-OH-D levels below 30 nmol/L. This observation was even more concerning because the data were collected at the beginning of the COVID-19 pandemic, which in Slovenia presented a huge burden on the healthcare system and resulted in high mortality among older adults. During the first wave of the pandemic in Slovenia (between March and May 2020), most deaths after infection with SARS-CoV-2 were NH residents [46]. The effects of the whole pandemic were catastrophic for this risky population. While the total death toll in Slovenia was about 10,000 people (about 500 per 100,000 people), over 3000 of these were NH residents (about 12,000 per 100,000 people) [55]. Considering that inadequate vitamin D status was hypothesized as a risk factor for high mortality in NH during the COVID-19 pandemic [56], the preliminary results of our study were used as support evidence for the preparation of vitamin D supplementation recommendations for physicians, which were published on the website of the Slovenian Endocrine Society [57]. While vitamin D supplementation was informally proposed, it is of particular importance to further investigate the prevalence of vitamin D deficiency in NH residents in detail. While low biosynthesis of vitamin D is expected in this population group due to decreased mobility and resulting low sun exposure, vitamin D status in older adults can also be affected by metabolic changes and impaired function of skin synthesis of vitamin D [58]. For example, a high prevalence of vitamin D deficiency has been reported even in cases of high sun exposure in the summer in older males living in a basic care unit in the tropics [59]. This was noted in more dark skin types, which were observed to produce less vitamin D compared to white skin types at normal sun exposure [60]. All things considered, there is evidence that NH residents would benefit from routine vitamin D supplementation to improve their vitamin D status [61,62]. In fact, vitamin D supplementation could be beneficial for various health conditions common in older adults, e.g., diabetes, hypertension, inflammation, and depression [63,64,65,66,67]. Considering the fact that foods, with the exception of, e.g., fatty fish, which are not abundantly consumed in Slovenia, are not good sources of vitamin D, supplementation of vitamin D would be beneficial, especially in the winter months when sun exposure is inadequate [29].

We also investigated vitamin B12 status; more than half of the participants had a low serum vitamin B12 concentration (<221 pmol/L). In males, the prevalence of suboptimal serum vitamin B12 concentration was slightly more marked than in females. This is in line with previous research, which highlighted older males as being more at risk for vitamin B12 deficiency, mostly due to absorption issues [68,69]. In Slovenian community-dwelling older adults, 10% of the population (12% males and 9% females) had a serum vitamin B12 concentration below 148 pmol/L, while the prevalence of a serum vitamin B12 concentration below 221 pmol/L was 46% [9]. Interestingly, there was a trend toward higher prevalence of low vitamin B12 status and deficiency observed in NH daycare center users, but statistical differences were not assessed. Our study’s results demonstrate that vitamin B12 should be screened on a larger scale in this population to ensure timely interventions where necessary. It should be noted that only about 1–2% of vitamin B12 is absorbed through passive diffusion via mucous membranes and the surface of the gastrointestinal tract, and the majority is absorbed through a receptor-mediated process through B12-specific proteins, intrinsic factors, and haptocorrin [70]. If the latter process is not functioning, which is common in older adults, particularly due to the prevalence of atrophic gastritis [71], the dietary intake of vitamin B12 cannot always provide the body with enough of this vitamin. In such cases, vitamin B12 intakes must be much higher in order to ensure sufficient absorption through passive diffusion pathways, while vitamin B12 can also be delivered non-orally, i.e., by subcutaneous injection or intravenous infusion. In cases where vitamin B12 absorption is not functional enough, a suitable method for supplementation is encouraged [70,72].

We should also note some limitations of the present study. Considering the pilot nature of the study, we used a rather small sample size (*n* = 37), meaning that the study results are not representative of the wider population in terms of using supplements and other exclusion criteria mentioned; however, we indicated several important dietary challenges that need to be addressed in larger studies. Another limitation is that all body height measurements were performed using a standard scale; future larger scale studies with older adults should also consider alternative body height measurements (prediction equations using ulna length) that could also be used in individuals with postural changes or other conditions that limit the use of standard measuring techniques. Additionally, data on medical conditions other than those referring to exclusion and inclusion criteria was not collected. Due to the small sample size, we were unable to report on the statistical differences between NH residents and NH daycare center users. Furthermore, since we did not separately record dietary intake data for users of daycare centers, we were unable to present specific dietary intake information for this subgroup categorized by food consumption from NH and other sources. It is important that future studies make this distinction to offer enhanced insights into dietary behaviors. Also, records from the NH menus would provide additional insights on what is offered within the scope of NH meal provision. In the study, we combined both nutritional intakes of macronutrients and the assessment of vitamin D and vitamin B12 with blood biomarkers, enabling us to obtain much better insights into the intake and status of micronutrients than solely investigating dietary intake. In relation to this, we need to mention that the assessment of vitamin B12 deficiency is commonly performed by using two biomarkers, often using criteria for elevated homocysteine (Hcy). Although high Hcy is very common in older adults due to various reasons, the prevalence of vitamin B12 deficiency in our population could be slightly overestimated because the data on Hcy were not available, which is a limitation of the study. We should also note that, considering inclusion criteria, our study did not provide insights about the proportion of food supplement users among NH residents. Future population studies should focus both on users/non-users of supplements, as well as include other blood biomarkers and body composition measurements (besides the body mass index, which was used in the present study), which would enable a better and more comprehensive understanding of nutritional status in this population. Larger scale studies should be designed using sophisticated modeling methods to estimate the usual long-term nutrient intakes, not only habitual nutrient intakes. This current study highlights that certain nutrients as well as micronutrients might be critical in this population; however, more thorough investigations with the inclusion of other important markers of nutritional status should be performed on a large, representative sample. Such a study should also focus on the intake of other nutrients, vitamins, and minerals. Furthermore, future larger studies should cover a wide variability of NHs, also taking into account the size and geographic location of the facility, with well-defined selection procedures.

Additionally, it is worth noting that the case study we have reported played a vital role in shaping the protocol for the larger study and securing funding for future research endeavors. Conducting nationally representative studies in high-risk populations poses significant challenges and financial burdens. In situations where researchers face a lack of supporting data to justify the necessity of such studies, as was the case in Slovenia, smaller pilot studies serve as valuable resources for collecting such data. The case study we have documented was instrumental in obtaining funding for the nationally representative NutriCare study (SRA Z3-3213), which promises to yield more comprehensive insights into this topic.

## 5. Conclusions

This pilot study highlighted that older adults in Slovenian nursing homes would require a comprehensive evaluation of nutritional challenges with a representative nation-wide study to enable appropriate public health interventions. A pilot study provided data to support the planning and conduct of such a study, including sample size calculations. Although this study was not powered to provide representative population-based epidemiological data, the results from a small sample indicated that the intake of certain nutrients as well as micronutrients require further attention. Adequate energy and protein daily intakes might be particularly critical, as well as the daily fat intake. A high prevalence of vitamin D deficiency and inadequate intake was indicated, so vitamin D supplementation should be considered in this population. Males were more at risk for vitamin B12 deficiency. To enable additional insights, large-scale studies would also need to explore other diet-related health risks, including muscle mass quantity and function, to provide insights on sarcopenia, which is often an overlooked and underestimated condition in this population.

## Figures and Tables

**Table 1:** Daily dietary intakes of energy and selected nutrients for males and females in nursing homes and nursing home daycare center users, compared to the reference values.

	Male (*n* = 13)			Female (*n* = 24)			
Macronutrient Intake	Median	P25	P75	Median	P25	P75	
Energy (kcal/day)	1637.4	1403.4	1982.1	1356.3	1272.0	1499.8	
Protein (g/day)	56.7	52.2	66.2	49.9	43.7	62.3	
g/kg body weight	0.6	0.5	0.9	0.8	0.5	0.9	
% TEI	13.5	12.7	15.2	15.2	13.1	17.7	
Carbohydrates (g/day)	207.5	157.3	237.0	165.6	121.1	193.7	
% TEI	52.6	45.6	53.6	50.2	42.9	52.3	
Fat (g/day)	66.7	50.5	76.2	51.1	43.7	60.2	
% TEI	32.6	30.8	39.9	35.0	31.6	37.7	
Vitamin B12 intake (µg/day)	2.3	1.9	3.0	2.0	1.6	2.9	
Vitamin D intake (µg/day)	1.7	1.4	2.3	1.5	1.0	2.8	
Compliance of intake with recommendations	Below DRV (%)	Above DRV (%)		Below DRV (%)	Above DRV (%)		DRV
Energy	92.3	7.7		95.8	4.2		2100/1700 (kcal/day) *
Protein	92.3	7.7		91.7	8.3		1 g/kg body weight
Carbohydrates	46.2	53.8		50.0	50.0		>50% energy intake
Fat	15.4	84.6		20.8	79.2		30% energy intake
Vitamin B12	92.3	7.7		87.5	12.5		4 µg/day
Vitamin D	100			100			20 µg/day

Notes: P—percentile; TEI—total energy intake; DRV—national dietary reference values for older adults [35]; * Adopted from national reference values for daily energy intake of older adult males (2100 kcal) and females (1700 kcal) with low physical activity.

**Table 2:** Body mass index, daily intakes, and compliance with the recommendations for nursing home residents and nursing home daycare center users.

S	NH Residents (*n* = 20)			NH Daycare Center Users (*n* = 17)		
Variable	Median	P25	P75	Median	P25	P75
Body mass index	29.9	25.0	30.6	28.6	26.2	29.8
Energy (kcal/day)	1467.8	1285.2	1792.3	1346.3	1297.0	1483.8
Protein (g/day)	50.6	42.9	57.2	58.5	49.8	69.3
g/kg body weight	0.7	0.5	0.8	0.8	0.6	0.9
% TEI	13.1	12.6	14.0	16.7	15.7	20.9
Carbohydrates (g/day)	206.5	165.9	233.7	151.3	116.0	176.3
% TEI	53.6	51.4	57.5	44.8	38.9	49.0
Fat (g/day)	50.8	45.8	72.2	55.2	45.5	65.8
% TEI	32.5	28.3	35.6	37.3	33.5	40.0
Daily vitamin B12 intake (µg/day)	2.2	1.8	2.8	2.1	1.6	3.0
Daily vitamin D intake (µg/day)	1.5	1.0	1.6	2.6	1.4	4.0
Compliance with recommendations	% below DRV	% above DRV		% below DRV	% above DRV	DRV
Energy	95	5		94	6	2100/1700 (kcal/day) *
Protein	100	0		82	18	1 g/kg body weight
Carbohydrates	15	85		82	18	>50% energy intake
Fats	35	65		0	100	30% energy intake
Vitamin B12	95	5		82	18	4 µg/day
Vitamin D	100			100		20 µg/day

Notes: NH—nursing home; P- percentile; TEI—total energy intake; DRV—national dietary reference values for older adults [35]; * Adopted from national reference values for daily energy intake of older adult males (2100 kcal) and females (1700 kcal) with low physical activity.

**Table 3:** Serum 25-OH-vitamin D and vitamin B12 concentrations and prevalence of deficiency, low serum 25-OH-vitamin D and vitamin B12 concentration and optimal status in males/females and nursing home residents/nursing home daycare center users.

		**25-OH-Vitamin D Concentration (nmol/L)**			**Prevalence (%)**			
	***n* (%)**	**Median**	**P25**	**P75**	**<30 nmol/L**	**30–50 nmol/L**	**50–75 nmol/L**	**>75 nmol/L**
All subjects	37	22.7	17.5	29.6	78.4	10.8	10.8	0
Male	13 (35)	21.3	17.5	27.3	92.3	7.7		0
Female	24 (65)	23.7	16.5	33.7	70.8	12.5	16.7	0
NH residents	20 (100)	17.6	14.6	20.3	100	0		0
NH daycare center users	17 (100)	29.7	23.9	42.4	52.9	23.6	23.5	0
	***n* (%)**	**Vitamin B12 concentration (pmol/L)**			**Prevalence (%)**			
		**Median**	**P25**	**P75**	**<148 pmol/L**	**148–221 pmol/L**		**>221 pmol/L**
All subjects	37	182.0	141.5	237.5	27.0	37.9		35.1
Male	13 (35)	164.0	142.0	231.0	30.8	38.5		30.7
Female	24 (65)	196.0	145.5	275.0	25.0	37.5		37.5
NH residents	20 (100)	185.5	151.5	297.5	20.0	40.0		40.0
NH daycare center users	17 (100)	182.0	141.0	223.0	35.3	35.3		29.4

Notes: NH—nursing home; P—percentile.

## Data Availability

The raw data supporting the conclusions of this article will be made available by the authors on request.
